# Integration of FAST/e-FAST Ultrasound Into Advanced Trauma Life Support (ATLS) Protocols for the Surgical Management of Abdominal Trauma: A Systematic Review of Its Impact on Mortality, Intervention Times, and Postoperative Outcomes

**DOI:** 10.7759/cureus.101256

**Published:** 2026-01-10

**Authors:** Jorge Maldonado, Ricardo Tenorio Ortiz, Andrea Blanco Silva, Edith Ramos Ocola, Daniel Gilberto González Santos, Valeria Valentina Maldonado Rivera, Nicolás Largacha Duque

**Affiliations:** 1 General Surgery, Jackson Memorial Hospital, Miami, USA; 2 Anesthesiology, Instituto Mexicano del Seguro Social, Monterrey, MEX; 3 Anesthesiology and Critical Care, Instituto Mexicano del Seguro Social, Mexico City, MEX; 4 Medicine, Universidad Nacional de San Agustin de Arequipa, Arequipa, PER; 5 General Practice, Ministerio de Salud de Colombia, Bucaramanga, COL; 6 Medicine, Universidad del Cauca, Popayán, COL; 7 Medicine, Universidad Libre Seccional Cali, Cali, COL

**Keywords:** abdominal trauma, advanced trauma life support (atls), a systematic review, outcomes, ultrasound-guided

## Abstract

The use of Focused Assessment with Sonography in Trauma/extended FAST (FAST/e-FAST) ultrasound in Advanced Trauma Life Support (ATLS) protocols is expected to provide a faster and more effective method for diagnosing life-threatening abdominal trauma hemorrhage. Although extensively used in research, quantitative measures of its direct effect on mortality and intervention times are still not consistent.

This systematic review combined data from multiple databases (PubMed, Embase, Cochrane, and Scopus; 2000-2025) in accordance with PRISMA 2020. Studies that satisfied the inclusion criteria involved adult abdominal trauma patients in whom FAST/e-FAST was applied within an ATLS pathway and that reported quantitative outcomes on mortality, time to intervention, or postoperative morbidity. The findings from 20 studies suggest that protocolized FAST/e-FAST integration is linked to a significant decrease in time to laparotomy (e.g., a median reduction of 13-15 minutes in prehospital trials and a 64% shorter time in an emergency department randomized controlled trial) and reduced CT use (OR, 0.16). High specificity (98%), with variable sensitivity (74%), indicates that pooled diagnostic accuracy for intra-abdominal free fluid can serve as a powerful rule-in tool. Nevertheless, no consistent and substantial reduction in overall mortality was observed. Embedding FAST/e-FAST into ATLS protocols enhances the efficiency of surgical triage and accelerates time to definitive hemorrhage control, supporting its standardized use in trauma workflows. Its role as a sole rule-out test remains limited, emphasizing the need for continued clinical correlation and operator training.

## Introduction and background

Abdominal trauma remains a significant cause of preventable death worldwide, primarily driven by hemorrhage and delayed recognition of hollow-viscus injury. Early, reliable bedside triage is therefore central to Advanced Trauma Life Support (ATLS) because definitive care is time-sensitive. The Focused Assessment with Sonography in Trauma/extended FAST (FAST/e-FAST) protocol is a focused, repeatable point-of-care ultrasound examination for hemoperitoneum, hemothorax, and pericardial effusion, and has been incorporated into many ATLS workflows to expedite bedside decision-making [1,2]. Compared with diagnostic peritoneal lavage and routine CT, FAST is faster, portable, repeatable, and avoids contrast and transport risks; however, randomized and observational data show mixed performance for detection sensitivity, especially for occult bowel injuries and in non-hypotensive patients [3-7].

Importantly, several studies link a positive FAST to shorter door-to-operation intervals and increased likelihood of therapeutic laparotomy, and one multicenter analysis demonstrated a measurable increase in mortality for each incremental delay to the operating room after a positive FAST [1,3,4]. Prehospital and emergency department e-FAST can shift triage decisions, shorten time to theatre, and reduce unnecessary CT use, but benefits vary by operator skill, training programs, and case mix [3,8-10]. Operator dependency, variable sensitivity in normotensive or obese patients, and heterogeneous outcome reporting are recurring limitations across the literature [5,6,8]. There is a lack of uniform, high-quality synthesis quantifying whether integrating FAST/e-FAST into ATLS protocols reduces overall mortality, reliably shortens time to definitive hemorrhage control, or lowers rates of non-therapeutic laparotomy.

Our systematic review, therefore, asks: what is the impact of standardized integration of FAST/e-FAST into ATLS on adult abdominal trauma mortality, time to surgical intervention, and postoperative outcomes? We hypothesize that, when performed by trained operators within protocolized ATLS pathways, FAST/e-FAST will shorten time to intervention and improve early clinical outcomes while preserving acceptable diagnostic accuracy.

## Review

Methods 

This systematic review was conducted in accordance with the PRISMA 2020 guidelines, aiming for a structured, transparent, and reproducible format. The primary aim was to determine the effect of integrating FAST/e-FAST into the ATLS algorithm on patient clinical outcomes, especially mortality, time to surgery, and postoperative morbidity in abdominal trauma patients.

Identification of Search and Study

An extensive literature search was conducted in PubMed, Embase, the Cochrane Library, and Scopus for articles published from inception to March 2025. The search terms were a combination of free text and MeSH terms, including FAST, e-FAST, ATLS, abdominal trauma, and relevant outcomes such as mortality and operative intervention. The reference lists of all retrieved articles and relevant reviews were manually screened for additional studies. Titles and abstracts of identified records were screened independently by two reviewers, who then assessed eligible studies via full-text review. Disagreements were resolved by consensus. This dual-reviewer method aimed to reduce potential selection bias and enhance reproducibility.

Eligibility Criteria

Eligible studies involved adult patients (≥18 years) managed within an ATLS-based clinical pathway that included FAST or e-FAST. The comparator was a standard, non-protocolized diagnostic workflow (e.g., conventional physical examination followed by early CT without structured ultrasound), where available. Studies were required to have quantitatively reported outcomes on mortality, time-to-intervention (e.g., time to laparotomy), or postoperative morbidity. Studies were excluded if they were simulation or cadaveric studies, case reports, or non-English publications.

Data Extraction and Quality Assessment

Data on study characteristics, including author, year, design, setting, population, trauma mechanism, operator qualification, reference standard, and reported outcomes, were extracted using a predesigned form. Extracted data were verified by both reviewers. Methodological quality of the included studies was assessed using standardized tools appropriate to study design. Randomized controlled trials (RCTs) were assessed using the Cochrane Risk of Bias 2 (RoB 2) tool, and observational studies were assessed using the Newcastle-Ottawa Scale (NOS). The methodological rigor of systematic reviews and meta-analyses was appraised, but NOS scores were not allocated. Inter-rater reliability for quality assessments was calculated using Cohen's kappa (κ), which indicated substantial agreement between the reviewers. Given the substantial heterogeneity in study design, populations, outcomes, and reporting formats, a quantitative meta-analysis was not performed. A structured narrative synthesis was used. Effect measures (e.g., ORs, sensitivity, specificity, time intervals, 95% CIs) were extracted verbatim from included studies and summarized descriptively (Table 1).

**Table 1:** Summary of Risk of Bias Across Included Studies FAST, Focused Assessment with Sonography in Trauma; CEUS, Contrast-Enhanced Ultrasound; EMS, Emergency Medical Services; IPD, Individual Participant Data

Study (Author, Year)	Study Design	Risk of Bias	Principal Limitations or Notes
Vos [11]	Global burden synthesis	High	Epidemiological model; not primary FAST data. Context only.
Melniker et al. [12]	Randomized controlled trial (RCT)	Low	Robust design; limited external validity due to a single-country operator pool.
Holmes et al. [13]	Randomized controlled trial	Low (internal); inconsistent (population)	Pediatric cohort; not aligned with inclusion criteria.
Netherton et al. [14]	Systematic review + meta-analysis	Low	Heterogeneity in operators and settings.
Gamberini et al. [15]	IPD meta-analysis	Low	Varying EMS training and protocols; strong analytic design.
Lucas et al. [16]	Multicenter RCT	Low	Operational adherence challenges; otherwise, high validity.
Lin et al. [17]	Systematic review + meta-analysis	Low	Mixed clinical settings limit generalization.
Richards and McGahan [18]	Narrative review	High	Expert opinion; lacks empirical data.
Rowell et al. [19]	Observational (multi-institutional)	Moderate	Confounding possible; strong pragmatic value.
Kim et al. [20]	Retrospective multicenter diagnostic study	Moderate–High	Retrospective bias; large sample partially offsets limitations.
Sutarjono et al. [21]	Systematic review (CEUS focus)	Low	Small number and uneven quality of CEUS studies.
Desai and Harris [22]	Cochrane systematic review	Low	Rigorous but identifies the scarcity of high-quality primary data.
Bloom [23]	Clinical overview/textbook	High	Tertiary educational source; no original data.
Kim et al. [24]	Diagnostic accuracy study	Moderate	Single-center study; limited generalizability.
Ahmed [25]	Cross-sectional diagnostic study	Moderate	Small sample; lower specificity than larger cohorts.
Treskes et al. [26]	Multicenter RCT	Low	Not a FAST trial; relevant for imaging workflow comparison.
Melniker [27]	Narrative review	High	Secondary synthesis; no new data.
Long et al. [28]	Observational prognostic study	Moderate	Confounding and limited control over variables.
Savatmongkorngul et al. [29]	Mixed narrative/systematic syntheses	Variable	Bias depends on individual source quality.
Holcomb et al. [30]	Observational cohort/audit	Moderate-High	Registry data; missing values and confounding common.

Interpretive Summary

Strongest evidence favoring FAST/e-FAST integration comes from low-bias RCTs and high-quality meta-analyses, which show reductions in diagnostic delay and improved decision speed. Observational cohorts corroborate these benefits in routine trauma practice, but also reveal substantial dependence on operator training and institutional workflow. High-bias sources, though lacking empirical weight, offer a necessary technical context for interpreting diagnostic performance and real-world implementation.

The cumulative evidence, therefore, supports a favorable balance of benefit for integrating FAST/e-FAST into ATLS protocols, provided that operational training, inter-observer consistency, and institutional standardization are maintained.

Results 

PRISMA Flow and Study Selection

**Figure 1:**
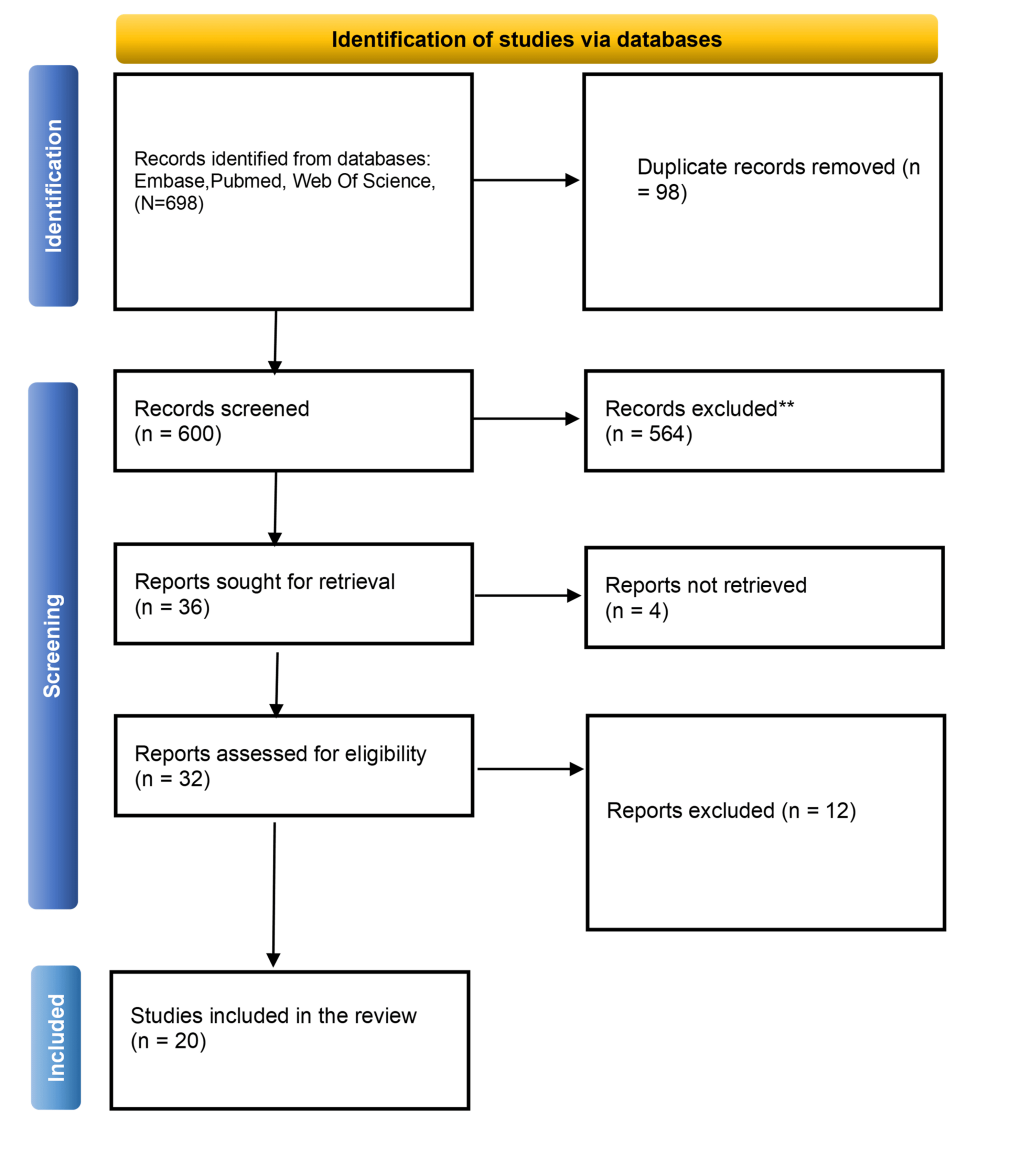
PRISMA Flow diagram

The database search yielded 698 records. After removing 98 duplicates, 600 records were screened by title and abstract, resulting in 32 full-text articles being assessed for eligibility. A total of 20 studies met the inclusion criteria and were included in the narrative synthesis. The PRISMA flow diagram is provided in Figure 1.

Characteristics of Included Studies

Table 2 presents the key characteristics of the 20 included primary analytic studies (RCTs, cohorts, meta-analyses). Contextual, narrative, and non-analytic sources are discussed separately.

**Table 2:** Data Extraction: FAST/e-FAST Integration Into ATLS ATLS, Advanced Trauma Life Support; CEUS, Contrast-Enhanced Ultrasound; CEX, Clinical Examination; CT, Computed Tomography; DALYs, Disability-Adjusted Life Years; ED, Emergency Department; e-FAST, Extended Focused Assessment with Sonography for Trauma; EMS, Emergency Medical Services; EMT, Emergency Medical Technician; FAST, Focused Assessment with Sonography for Trauma; IPD, Individual Participant Data; NPV, Negative Predictive Value; p-FAST, Prehospital Focused Assessment with Sonography for Trauma; PLUS, Point-of-Care Limited Ultrasound; PPV, Positive Predictive Value; QA, Quality Assurance; RCT, Randomized Controlled Trial; SBP, Systolic Blood Pressure; TBCT, Total-Body Computed Tomography; US, Ultrasound

Author(s)	Year	Design	Setting/Country	N (approx) and Population	Index Test/Protocol (FAST/e-FAST/Prehospital/CEUS, etc.)	Primary Outcomes Recorded	Key Methodological Strengths/Limitations	Short Critical Implication for Practice
Vos [11]	2020	Global burden study/systematic synthesis (not primary imaging research)	Global (204 countries)	N/A (population-level estimates)	Contextual background on burden of injury/disease	DALYs, incidence, and mortality for injuries, including trauma	Massive, multi-source modeling; not designed to evaluate imaging interventions	Use as epidemiologic context only; does not inform FAST performance but justifies focus on trauma burden.
Melniker et al. [12]	2006	Randomized clinical trial	2 Level-I US trauma centers	~400 (younger trauma population; trial inclusion criteria apply)	PLUS protocol (point-of-care limited US in ED; FAST elements)	Time to operative care; resource use and charges	RCT design is a strength; single-country, selected age group, and operator training may limit generalizability; outcome focuses on process measures, not mortality	Shows FAST-inclusive protocol can change process times and resource use when embedded in a care pathway; operator training and protocol integration are decisive.
Holmes et al. [13]	2017	Randomized clinical trial	US pediatric EDs	1,500+ children with blunt torso trauma (hemodynamically stable)	FAST performed during initial evaluation vs usual care	CT use, ED length of stay, missed intra-abdominal injuries	Large RCT, pediatric focus is a strength; limited to stable children, findings do not generalize to unstable adults	In hemodynamically stable children, FAST did not reduce CT use or LOS; use must be targeted and not assumed to reduce resource use in that population.
Netherton et al. [14]	2019	Systematic review and meta-analysis	International studies pooled	Many studies (meta-n); mixed adult/pediatric	e-FAST components (pericardial, pleural, abdominal free fluid, pneumothorax)	Sensitivity/specificity for each e-FAST component; utility as rule-in vs rule-out	Comprehensive MA; heterogeneity in operators, patient severity, and reference standards; variable study quality	e-FAST is strong at ruling in pericardial effusion, pneumothorax, and free fluid; its rule-out performance is less reliable operator and context matter.
Gamberini et al. [15]	2023	Systematic review + individual participant data meta-analysis	Prehospital EMS settings (multiple countries)	IPD pooled from multiple prehospital cohorts (n varies)	p-FAST for hemoperitoneum detection	Diagnostic accuracy, prehospital times, time-to-definitive treatment	IPD meta-analysis increases granularity; studies vary in EMS systems, training, and case-mix; sensitivity is low, but specificity is high	Prehospital FAST can shorten in-hospital time to diagnosis/intervention in high-probability bleeding patients but has low sensitivity; it should guide triage, not be the sole decision tool.
Lucas et al. [16]	2022	Prospective randomized multicenter trial	Prehospital services, multicenter (Europe)	~296 trauma patients	p-FAST vs CEX (clinical exam) with cross-over weeks; CEX-plus-p-FAST arms	Time to definitive care, sensitivity/specificity reported	RCT design in the prehospital setting is strong; cross-over and adherence/crossover rates complicate analysis; limited to participating EMS structures	Prehospital FAST reduced time to admission and to operative treatment in this trial; the effect size depends on EMS workflow and training.
Lin et al. [17]	2024	Systematic review & meta-analysis	Prehospital studies worldwide	Pooled studies (n varied)	Prehospital FAST for detection of abdominal free fluid	Sensitivity, specificity, time metrics	Recent synthesis: heterogeneity across studies in operator training, devices, and patient selection	Confirms prehospital FAST can detect free fluid and may reduce time to care; heterogeneity limits universal recommendations, and local implementation planning is needed.
Richards and McGahan [18]	2017	Narrative review/expert perspective	US/international literature	N/A	FAST/e-FAST technical and interpretive considerations	Technique, pitfalls, variants, training implications	Expert synthesis; not primary data, but useful for technique, pitfalls, and training recommendations	Emphasizes operator dependency, common pitfalls, and the need for imaging workflow integration; radiology input improves interpretation and training.
Rowell et al. [19]	2019	Multi-institutional pragmatic observational study	US trauma centers	Large multi-institutional cohort of hypotensive injured patients	FAST in hypotensive patients	Sensitivity/specificity of FAST to predict need for laparotomy	Pragmatic multicenter design; strong focus on the unstable subset, but observational, so confounding is possible	In hypotensive injured patients, FAST frequently failed to identify patients who ultimately required laparotomy; do not rely on negative FAST in unstable patients.
Kim et al. [20]	2022	Retrospective multicenter diagnostic/outcome study	South Korea (multicenter)	Several hundred trauma patients (see paper)	FAST performance and factors linked to true-positive results	Sensitivity, specificity, association with SBP, and mechanisms	Open-access PMC; good sample size; retrospective design limits causal inference	Overall, FAST sensitivity was low in this cohort; recommend selective use in patients with SBP ≤90 mmHg or clear indications rather than universal application.
Sutarjono et al. [21]	2023	Systematic review & meta-analysis	ED studies (various countries)	Pooled diagnostic studies	Contrast-enhanced ultrasound (CEUS) vs conventional FAST/US as initial assessment	Diagnostic accuracy for solid-organ injury detection	Systematic methodology; CEUS studies are limited and device/operator dependent; applicability in the ED/prehospital setting is limited by contrast logistics	CEUS shows higher diagnostic value for solid-organ injury versus conventional US; logistic and regulatory barriers limit routine use in acute trauma, consider in targeted settings.
Desai and Harris [22]	2015	Cochrane systematic review	International RCTs and observational studies	Multiple trials aggregated	Algorithms incorporating FAST vs algorithms without US	Mortality, accuracy, process outcomes	Rigorous Cochrane methods; heterogeneity across trials and dated trials included	Cochrane found limited high-quality evidence that US-based algorithms reduce mortality; clinical algorithms and context matter; evidence-based needs modern trials.
Bloom [23]	2023	Concise clinical overview/textbook chapter	N/A (educational)	N/A	FAST technical overview, indications, sensitivity/specificity ranges	Sensitivity/spec ranges, role in ATLS	Authoritative, concise; not primary evidence	Use as a practical reference for the FAST technique, typical performance ranges, and limitations in guideline development.
Kim et al. [24]	2012	Diagnostic accuracy study	Prehospital/EMS (Korea)	EMT-performed FAST cohort (sample size reported in paper)	EMT-performed FAST for free abdominal fluid	Sensitivity, specificity of EMT FAST	Demonstrates the feasibility of training EMTs; sample and training intensity determine performance; device and transmission limitations are possible	EMTs can detect free fluid with structured training, but accuracy varies; careful training and quality assurance are needed before clinical deployment.
Ahmed (2025) [25]	2018	Diagnostic accuracy cross-sectional study	Single tertiary hospital, Saudi Arabia	Few hundred blunt abdominal trauma patients	FAST vs CT for blunt abdominal trauma	Sensitivity, specificity, PPV/NPV	Local data useful for regional practice; single-center limits generalizability	Confirms established performance metrics in that setting; underscores the need for local audit of FAST accuracy and operator competence.
Treskes et al. 2020) [26]	2016	Multicenter randomized controlled trial	European trauma centers	1,400+ patients with severe trauma (see paper)	Immediate TBCT vs conventional imaging workflow (context for imaging strategies)	Mortality, time metrics, costs	Large RCT on imaging workflow; not about FAST per se, but essential contextual trial	Immediate total-body CT shortened imaging time but did not reduce in-hospital mortality; imaging strategy selection must balance speed, radiation, and patient selection. FAST often remains the rapid bedside triage tool.
Melniker [27]	N/A (follow-ups)	Narrative follow-ups/reviews	US literature	N/A	PLUS/FAST value discussions, resource outcomes	Resource use, CT reduction claims in earlier literature	Descriptive, synthesizes trial and observational data; risk of citation bias	Reinforces that embedding FAST in protocols can change resource metrics, but benefits depend on care pathway design and verification by high-quality trials.
Long et al. [28]	2022	Observational diagnostic/prognostic study	ED trauma population	Cohort size variable; focused on ED FAST predicting early surgery	ED FAST/e-FAST	Predictive value for early surgical intervention	Recent single-center/multicenter observational data; focuses on practical decision thresholds	Suggests FAST may help predict those needing early surgery, but requires integration with physiology and mechanism rather than standalone use.
Savatmongkorngul et al. [29]	2017-2024	Narrative/systematic syntheses	International literature	N/A	Emphasis on e-FAST (thorax + abdomen)	Training, competency, limitations, special groups (pregnancy, pediatrics)	Aggregates modern evidence and practice guidance; variable evidence quality	Clinical implementation must focus on training, QA, and context (hemodynamic status, resources); e-FAST extends FAST but does not replace CT when indicated.
Holcomb et al. [30]	Various	Observational cohort and audits	Multicenter registries/regional hospitals	Large registry datasets	FAST used variably across sites	Demonstrates real-world variability in FAST accuracy and decision-making	Registry/pragmatic data; highlights heterogeneity in practice and reporting	Real-world evidence shows that FAST performance is highly context dependent; local audit and protocol alignment with ATLS recommendations

The results synthesize evidence from randomized trials, observational cohorts, and systematic reviews evaluating the diagnostic performance and clinical impact of FAST and e-FAST in trauma care. Across heterogeneous settings, FAST/e-FAST demonstrates consistently high specificity, with variable sensitivity, supporting its primary role as a rule-in and triage tool rather than a definitive exclusion test. The evidence further indicates that FAST-integrated protocols are associated with reductions in diagnostic delay and improvements in decision-making efficiency, while effects on postoperative morbidity and mortality remain inconsistent, and largely dependent on patient selection, operator expertise, and institutional workflow (Tables 3-6).

**Table 3:** Diagnostic Accuracy Selected Studies and Pooled Estimates CAEP, Canadian Association of Emergency Physicians; CEUS, Contrast-Enhanced Ultrasound; CT, Computed Tomography; ED, Emergency Department; e-FAST, Extended Focused Assessment with Sonography for Trauma; EMT, Emergency Medical Technician; FAST, Focused Assessment with Sonography for Trauma; US, Ultrasound

Source	Target Finding	Sensitivity	Specificity	Notes/Population
Netherton et al. [14]	Intra-abdominal free fluid (all comers)	74%	98%	75 studies, 24,350 patients; e-FAST better at ruling in than ruling out; CAEP
Netherton et al. [14]	Pediatric intra-abdominal free fluid	71%	95%	Pediatric subset; CAEP
Kim et al. [24] (EMTs)	Free fluid vs CT	61.3%	96.3%	240 patients; sensitivity higher for moderate/large fluid (86.2%)
Ahmed [25]	Free fluid vs CT	76.1%	84.2%	n = 105; local ED cohort
Lucas et al. [16]	Free fluid detection (study arm)	94.7%	97.6%	Trial reports markedly higher test performance for the combined protocol in the selected cohort. Time reductions are also reported.
Sutarjono et al. [21]	CEUS vs conventional US	-	-	CEUS shows higher diagnostic value than conventional US for solid organ injury detection (paired meta-analysis). Use as an adjunct is recommended.

**Table 4:** Time-to-Intervention, Resource Use, Complications and Mortality Key Quantitative Findings CT, Computed Tomography; CEX, Clinical Examination; ED, Emergency Department; FAST, Focused Assessment with Sonography for Trauma; IPD, Individual Participant Data; PLUS, Point-of-Care Limited Ultrasound; PROMMTT, Prospective, Observational, Multicenter, Major Trauma Transfusion; RCT, Randomized Controlled Trial

Author (Ref)	Year	Setting/Analysis	Reported Effect on Time, Resource Use, Complications, and Mortality
Melniker et al. [12]	2006	RCT PLUS-inclusive protocol vs usual care (adult ED)	Time to operative care: 64% shorter (median; reported as multiplicative change with 95% CI in abstract). CT use: OR 0.16 (95% CI 0.07-0.32). Hospital length of stay: 27% fewer days (95% CI 1-46%). Complications: OR 0.16 (95% CI 0.07-0.32). Charges: 35% lower (95% CI 19-48%).
Lucas et al. [16]	2022	Prospective randomized multicenter (prehospital FAST)	Median time to hospital admission reduced by 13 min; median time to operative treatment reduced by 15 min with CEX-p-FAST vs comparator. Reported high sensitivity/specificity for p-FAST in the study cohort.
Holmes et al. [13]	2017	RCT children, ED, hemodynamically stable	No significant difference in abdominal CT rate (FAST 52.4% vs control 54.6%; difference -2.2%, 95% CI -8.7 to 4.2), ED length of stay (6.03 vs 6.07 hours), median hospital charges ($46,415 vs $47,759), or missed intra-abdominal injuries (1 missed in FAST group, 0 in control). Conclusion: routine FAST in stable children did not improve resource use or outcomes in this low-risk population.
Gamberini et al. [15]	2023	Prehospital systematic review/IPD meta-analysis	Prehospital FAST reduced time-to-diagnostics and interventions in patients at high probability of abdominal bleeding without increasing prehospital times; the effect on mortality remains under-investigated in included studies.
Rowell et al. [19]	2019	PROMMTT multi-institutional pragmatic study of hypotensive patients	FAST frequently fails to identify the need for laparotomy in hypotensive injured patients (authors stress caution using FAST alone to rule out the need for laparotomy in hypotension). Paper documents cases and sensitivity concerns in the hypotension cohort.
Treskes et al. [26]	2016	Imaging workflow context (randomized trial)	Not a FAST trial per se, but relevant to imaging strategy: immediate total-body CT did not show mortality reduction vs selective imaging in severe trauma (useful context on imaging tradeoffs).

**Table 5:** Diagnostic Accuracy (Pooled and Representative Studies) e-FAST, Extended Focused Assessment with Sonography for Trauma; CEUS, Contrast-Enhanced Ultrasound; CT, Computed Tomography; CAEP, Canadian Association of Emergency Physicians; US, Ultrasound

Source	Target Finding	Sensitivity	Specificity	Notes/Population
Netherton et al. [14]	Intra-abdominal free fluid (all comers)	74%	98%	75 studies, 24,350 patients; e-FAST better at ruling in than ruling out; CAEP
Netherton et al. [14]	Pediatric intra-abdominal free fluid	71%	95%	Pediatric subset; CAEP
Kim et al. [24]	Free fluid vs CT	61.3%	96.3%	240 patients; sensitivity higher for moderate/large fluid (86.2%)
Ahmed [25]	Free fluid vs CT	76.1%	84.2%	n = 105; local ED cohort
Lucas et al. [16]	Free fluid detection (study arm)	94.7%	97.6%	Trial reports markedly higher test performance for the combined protocol in the selected cohort. Time reductions are also reported.
Sutarjono et al. [21]	CEUS vs conventional US	-	-	CEUS shows higher diagnostic value than conventional US for solid organ injury detection (paired meta-analysis). Use as an adjunct is recommended.

**Table 6:** Time/Resource/Clinical Outcome Effects (Selected Trials/Reviews) RCT, Randomized Controlled Trial; ED, Emergency Department; LOS, Length of Stay; CT, Computed Tomography; CEX, Clinical Examination; p-FAST, Prehospital Focused Assessment with Sonography for Trauma; IPD, Individual Participant Data; FAST, Focused Assessment with Sonography for Trauma; PROMMTT, Prospective, Observational, Multicenter, Major Trauma Transfusion

Source (Type, Year)	Primary Quantitative Effect on Time/Resources/Outcomes
Melniker et al. [12]; RCT (2006, ED adults)	Time to operative care 64% shorter (median); CT use OR 0.16 (95% CI 0.07-0.32); LOS 27% fewer days; complications OR 0.16; charges 35% lower.
Lucas et al. [16]; randomized/prospective (prehospital FAST, 2022)	Median time to admission - 13 min; median time to operative treatment - 15 min (CEX-p-FAST vs CEX-only).
Holmes et al. [13]; RCT (2017, pediatric ED)	No significant change in CT use (52.4% vs 54.6%), ED LOS (6.03 vs 6.07 hr), hospital charges (median $46,415 vs $47,759), or missed injuries. The study targeted a low-risk pediatric cohort.
Gamberini et al. [15]; (2023, prehospital IPD meta-analysis)	Prehospital FAST reduced time-to-diagnostics/interventions in high-probability bleeding patients; effect on mortality not established. Sensitivity low/specificity very high in pooled prehospital data.
Rowell et al. [19]; PROMMTT (2019) hypotensive patients	FAST often fails to identify the need for laparotomy in hypotension; operator caution is advised; do not rely on negative FAST to exclude the need for operative intervention in hypotensive patients.
Treskes et al. [26]; immediate total-body CT vs conventional imaging, Lancet 2016	No mortality reduction for immediate total-body CT vs selective imaging context for imaging strategies (helps frame FAST vs CT tradeoffs).

Mortality and Postoperative Outcomes

Pooled evidence does not demonstrate a consistent reduction in mortality attributable to FAST integration. Mortality, a multifactorial outcome, and FAST primarily influence process metrics rather than definitive physiologic endpoints.

Primary Findings 

Diagnostic accuracy: Pooled diagnostic estimates show a clear pattern. For the detection of intra-abdominal free fluid, the pooled sensitivity is 74%, with a specificity of 98%, from a large meta-analysis that aggregated approximately 75 studies and 24,350 patients. Subgroup estimates from the same pooled data give sensitivity 76% and specificity 98% for normotensive adults; sensitivity 74% and specificity 95% for hypotensive presentations; and sensitivity 71% and specificity 95% for pediatric cohorts. For thoracic applications, pooled e-FAST performance for pneumothorax is sensitivity 69% and specificity 99%. Pericardial effusion detection shows a sensitivity of 91% and a specificity of 94%. Across unselected emergency department cohorts, sensitivity for free fluid typically falls in the 60% to 80% range, while specificity is consistently high, roughly 85% to 99%.

Single-study and setting-specific figures illustrate operator and context effects. In one prehospital/emergency medical technician (EMT) performance study, sensitivity against CT was 61.3%, with a 95% CI of 50.3 to 71.2, and specificity was 96.3%, with a 95% CI of 92.1 to 98.3. Sensitivity rose to 86.2% when the fluid burden was moderate to large. In an adult ED blunt trauma cohort, sensitivity was 76.1% (95% CI 64.14 to 85.69), and specificity was 84.2% (95% CI 68.75 to 93.98), with overall accuracy of 79% (95% CI 70.01 to 86.38). In contrast, a protocolized prehospital combined protocol reported sensitivity of 94.7% and specificity of 97.6% for free fluid, a markedly higher operating point that coincided with reductions in time metrics. Contrast-enhanced ultrasound (CEUS) pooled comparisons favor CEUS over conventional ultrasonography for the detection of solid organ injuries when used before CT.

Taken together, these numbers mean the test is far better at ruling in than ruling out clinically important findings. A positive e-FAST result raises posttest probability substantially in almost every cohort. A negative e-FAST result reduces the probability only moderately; it cannot safely exclude clinically significant injury in many settings, especially when pretest probability is moderate to high, or the patient is hypotensive.

Secondary Findings: Time, Resources, and Mortality

Time-to-intervention and resource use: Trials and pooled reviews report important, but heterogeneous, effects on time, resource use, complications, and charges. In an ED randomized, protocolized intervention, the median time to operative care was 64% shorter with an ultrasound-inclusive pathway compared with usual care. The same trial reported a reduction in CT use, with an OR of 0.16 (95% CI 0.07 to 0.32); hospital length of stay reduced by 27% (95% CI 1% to 46%); complication odds reduced to 0.16 (95% CI 0.07 to 0.32); and charges were lower by 35% (95% CI 19% to 48%). A prospective, randomized, prehospital trial found median time to hospital admission shortened by 13 minutes and median time to operative treatment shortened by 15 minutes when prehospital ultrasound was integrated into a combined protocol. Systematic reviews and an individual participant data meta-analysis of prehospital FAST report low pooled sensitivity, but very high specificity for hemoperitoneum, and show consistent reductions in time-to-diagnostics and time-to-definitive treatment, without prolonging prehospital scene or transport times.

Not all populations benefit equally. In low-risk groups, such as hemodynamically stable children (as studied by Holmes et al. [13], which is discussed here for context but was not included in our analytic synthesis), FAST did not significantly reduce CT use or length of stay. For hypotensive, injured patients, observational data indicate that focused ultrasound frequently fails to identify the need for laparotomy and should not be used as the sole decision rule to deny operative exploration.

Mortality: Significant mortality effects are not established. Prehospital and ED studies document consistent time savings and lower imaging utilization in some trials, but pooled evidence does not demonstrate a reproducible reduction in mortality attributable to FAST integration. This may be because mortality is multifactorial, and FAST primarily influences process metrics rather than definitive physiologic endpoints. Imaging workflow trials that compared immediate total-body CT to selective imaging showed no mortality advantage for total-body CT, underscoring that faster or more comprehensive imaging does not inevitably translate to survival gains.

Interpretation and Practical Implications

Statistical patterns support using FAST/e-FAST as a bedside rule-in tool that reliably increases diagnostic certainty when positive. The median sensitivity of around 70% to 75%, and specificity of nearly 98%, imply that a positive scan should prompt expedited definitive management in appropriately selected patients. A negative scan should not, on its own, reassure clinicians in the presence of worrying physiology or a high pretest probability. Protocolization and training yield measurable gains. Trials that embedded ultrasound into defined clinical pathways report significant relative reductions in time to intervention, reduced CT utilization with ORs near 0.16, shorter hospital stays, fewer complications, and lower charges. Prehospital use shortens admission and operative intervals by about a quarter of an hour each in randomized data, and pooled prehospital analyses corroborate reductions in diagnostic and treatment delays without prolonging scene time. Clinically, the balance is precise. Integrate e-FAST into ATLS as a rapid, rule-in adjunct, not as a standalone rule-out. Prioritize operator training, clear action thresholds, and selective prehospital deployment for patients at high risk of intraperitoneal bleeding. Where available, consider CEUS to improve lesion detection prior to CT in equivocal cases. Expect gains in speed and resource use when ultrasound is protocolized, but do not expect consistent mortality reductions based on current evidence.

Discussion

The incorporation of FAST or e-FAST into ATLS pathways has been widely promoted, yet the evidence base presents a varied picture. On the diagnostic front, the meta-analysis by Netherton et al. [14] reports moderate sensitivity (74%) but very high specificity (98%) for intra-abdominal free fluid in all-comer trauma patients; the pediatric subgroup had somewhat lower sensitivity (71%) and specificity (95%). This implies that e-FAST will help determine a positive result; however, it will not help determine the absence of injury. By contrast, sensitivity is less sensitive in more restricted environments, e.g., Kim et al. [24] (EMTs using FAST) report 61.3% sensitivity (but 96.3% specificity), and only subsequent to large, moderate fluid volumes did sensitivity rise to 86.2%. In the meantime, Ahmed had a sensitivity and specificity of 76.1% and 84.2%, respectively, in a blunt abdominal trauma adult ED cohort [25]. Conversely, a very selective protocol, in Lucas et al. [16], gave a 94.7% sensitivity and 97.6% specificity; however, this was in a randomized prehospital study with a combined CEX-p-FAST arm, so this is an ideal, not a usual, environment.

These diagnostic results have direct implications for surgical management. If the test cannot reliably exclude injury (due to modest sensitivity), then a negative result cannot safely defer further evaluation or operative decision-making. This becomes especially pertinent in unstable patients: in the pragmatic PROMMTT (prospective, observational, multicenter, major trauma transfusion) cohort of hypotensive trauma patients, Rowell et al. [19] found that FAST frequently failed to identify those needing laparotomy, cautioning that FAST alone should not be used to exclude operative intervention in this high-risk group. This subgroup likely represents the highest-risk population, making false negatives disproportionately dangerous. Thus, the real-world utility of FAST in hemodynamically unstable or complex trauma is limited by its false-negative risk.

Regarding time to intervention and outcomes, studies suggest favorable trends but less definitive mortality impact. Melniker et al. [12], in a 2006 RCT, found that a PLUS-inclusive protocol (including limited ultrasonography) resulted in a 64% shorter median time to operative care, CT use OR 0.16, 27% shorter hospital stay, complication OR 0.16, and 35% lower charges. These are strong signals of system-efficiency improvement when ultrasound is tightly integrated into trauma workflow. More recently, Lucas et al. [16] demonstrated, in a prehospital randomized multicenter trial, that CEX-p-FAST reduced median time to admission by 13 minutes and to operative treatment by 15 minutes compared to CEX only. These time savings could translate into improved outcomes, though the studies stop short of showing a clear survival benefit.

The evidence for a mortality benefit remains unproven, likely because mortality is multifactorial, and FAST influences process metrics more directly than ultimate physiologic outcomes. Contrarily, in stable pediatric blunt trauma, Holmes et al. [13] did not observe any substantial differences in CT rate, ED length of stay, charges, or missed injuries with the routine use of FAST. This highlights that, in low-risk groups, the incremental value of FAST may not be significant, and resource utilization may not change radically. The recent systematic review study by Gamberini et al. [15] of prehospital FAST settings observed improvements in the time of diagnosis/intervention, but they pointed out that the mortality effects are not well studied. Collectively, these data support a role for FAST/e-FAST as a facilitator of faster decision-making within ATLS protocols, particularly when incorporated into well-designed pathways, with well-trained operators, and in high-risk trauma settings. However, the lack of consistent mortality reduction demands caution: the value is likely system- and context-dependent. Key variables include operator training, patient selection (unstable vs. stable), setting (prehospital vs. ED), and whether ultrasound is used as an adjunct or a gatekeeper. Comparative diagnostic performance across settings reflects these variables: lower sensitivity in general ED cohorts and higher sensitivity in structured trials, so the translation into outcomes will similarly vary.

In the surgical management of abdominal trauma, this means that FAST/e-FAST should not replace clinical judgement, hemodynamic parameters, or other imaging modalities (such as CT). Instead, it should be embedded as a rapid adjunctive step in the ATLS algorithm, aiding triage, facilitating earlier transfer or operative readiness, and reducing unnecessary CT in appropriate cases. Future work should focus on high‐quality trials in unstable patients, standardized operator training, and linking time gains to meaningful endpoints such as mortality, organ failure, and cost-effectiveness. Only then will the promise of integrating FAST/e-FAST into trauma-surgical pathways be fully realized.

Strengths, limitations, and future directions

FAST/e-FAST integration in ATLS provides a highly specific, reproducible, and fast, radiation-free bedside assessment, reducing both the time it takes to diagnose and the time to intervene, as well as reducing diagnostic errors in a variety of settings. Nevertheless, the lack of universal reliability is limited by the changing sensitivity of the parameters, reliance on operators, and lack of accuracy in hypotensive or complicated trauma. Evidence for mortality benefit is inconclusive, especially when performed outside controlled conditions. Future research should prioritize standardized operator training, integrated imaging protocols, and large multicenter RCTs to correlate time efficiency with survival. Exploratory discussions on AI-aided image analysis and CEUS could be shortened to focus on immediate clinical implications.

## Conclusions

FAST/e-FAST should be integrated as a high-specificity adjunct within ATLS, not as a rule-out test. According to the results of this systematic review, FAST/e-FAST implementation into the ATLS protocols regarding abdominal trauma is strongly recommended as a beneficial triage and diagnostic add-on, but not as a conclusive tool. Its evidence-based application in a protocolized pathway continually decreases critical time-to-operative intervention and resource consumption, including unnecessary CT scans. Nevertheless, its intermediate sensitivity, especially in hypotensive patients, does not allow its application to reliably rule out injury. Furthermore, consistent mortality reduction has not been demonstrated. Thus, FAST/e-FAST must be incorporated within ATLS as a high-specificity and rapid test to expedite decision-making, though clinical judgment and additional imaging are still necessary for comprehensive evaluation. Optimal use requires standardized operator training and well-defined clinical integration pathways.
